# Editorial: Advances in the development of functional biomaterial nanosystem in tumor therapy and tissue regeneration

**DOI:** 10.3389/fbioe.2023.1195089

**Published:** 2023-04-04

**Authors:** Liangliang Dai, Xiaojiao Li, Zhang Yuan, Jinghua Li

**Affiliations:** ^1^ Xi’an Key Laboratory of Stem Cell and Regenerative Medicine, Institute of Medical Research, Northwestern Polytechnical University, Xi'an, China; ^2^ The First Affiliated Hospital of Xi’an Jiaotong University, Xi'an, China; ^3^ School of Medical Technology and Engineering, Henan University of Science and Technology, Luoyang, China

**Keywords:** functional biomaterial nanosystem, tumor therapy, tissue regeneration, drug delivery, bioimaging, surface and interfaces, nanomedicine

Benefiting from the fast development of nanotechnology and prominent advantages (e.g., biosafety, drug/gene storage capability, easy functionalization, etc.) of biomaterials composed of various organic or inorganic materials (such as proteins, polymer, silica-based nanoparticles, iron or gold nanoparticles), biomaterials are frequently used to constructed functional nanosystems integrated with abundant desired traits, such as simultaneous diagnosis and targeted cancer therapy, stimuli-responsive drug release, wound healing and tissue regeneration for tumor therapy, anti-inflammatory, tissue engineering and diseases diagnosis in recent years. The multidisciplinary attempts led to the development of several exciting clinically approved nanotherapeutics. In this Research Topic, the interdisciplinary researchers have exhibited the latest advances and achievements in functional biomaterial nanosystems for cancer treatment and tissue regeneration from different perspectives.

The primary inflammation process is intimately linked to oxidative stress, and blocking oxidative stress mediated by antioxidants may be beneficial for reducing inflammation (Li et al., 2018). However, a new powerful antioxidant agent with strong ROS-scavenging capability remains to be developed. Dou et al. construct a ferrite and ceria co-engineered mesoporous silica nanoparticles antioxidant agent though a facile metal Fe/Ce-co-doping approach in the MSN framework, which exhibited an excellent efficiency in scavenging reactive oxygen species (ROS). Furthermore, this nanosystem significantly attenuate ROS-induced inflammation and switch macrophages from a pro-inflammatory M1 phenotype toward an anti-inflammatory M2 phenotype.

Since the drug trapped in the solid matrix of the phase change materials (PCMs) could be released rapidly along with the melted PCMs (Qiu et al., 2020), PCMs have been widely used in smart drug delivery. A review by Bao et al. summarizes the latest developments on PCMs as smart gate-keepers for anti-tumor applications.

It is believed that chemodynamic therapy (CDT) combined with photothermal therapy (PTT) is a new therapeutic method to generate ROS or convert light energy into hyperthermia to inhibit cancer cell proliferation (Lv et al., 2021), and this therapeutic method revealed a prospective application in triple-negative breast cancer (TNBC) chest wall metastasis. However, the development of near-infrared light-responsive nanomaterials for CDT and PTT is a promising platform but still challenging in biomedicine. As Fe atoms embedded nitrogen-doped carbon (Fe-N-C) nanomaterials could act as single atom nanozymes (SAzymes) to achieve artificial enzyme-catalyzed reactions (Fu et al., 2018; Jiao et al., 2020), Qian et al.thus synthesized a Fe-N-C single atom nanozyme for enhanced TNBC therapy *via* CDT/PTT. These results demonstrates that the construction of Fe-N-C SAzymes could impair mitochondrial OXPHOS, promote glycolysis and generate ROS by decomposing H2O2 into ·OH, eventually induce tumor cells apoptosis.

Low-temperature photothermal therapy (PTT) mainly refers to the induction of tumor cell apoptosis under low power laser irradiation (Jung et al., 2016; Xia et al., 2021), which reduces the damage to normal tissue cells, however, it may not be able to achieve a satisfactory anti-tumor therapeutic effect due to the existence of molecular chaperones and heat shock proteins (HSPs). Therefore, combining low-temperature PTT with the reduction of HSPs can not only inhibit the protective effect of HSPs on tumor cells, but also improve the sensitivity of tumor cells to thermal stimulation, which is crucial in enhancing the efficacy of low-temperature PTT. Xin et al. review the progress of nanomaterial-mediated low-temperature PTT mainly in terms of inhibiting the expression of HSPs and provides an outlook of the translation of this strategy into real clinical applications.

Great advantages were shown for polymer nanocomposites (NCs) for cancer diagnosis/treatment due to their unique properties (e.g., eco-friendly nature, design capacity, cost-effectiveness, and facile production) (Feldman, 2019). EL-Sherbiny et al. aimed at constructing the potent anticancer NC composed of chitosan nanoparticles, curcumin and eugenol and evaluating its anticancer activity in depth against colorectal adenocarcinoma and breast adenocarcinoma cells. These findings suggest the usefulness and effectiveness of NCs composed of multi-natural molecules.

The specific recognition, low immunogenicity, and low cytotoxicity of aptamers make them themselves well suited for the diagnosis and treatment of tumors (Wang et al., 2022). Particularly, the active targeting of aptamers can more accurately select target cells to assist in the controlled and continuous transfer of therapeutic molecules [drugs (Go et al., 2021) and peptides (Kim et al., 2015)] using AuNPs and improve the uptake of therapeutic molecules by the cells. A review by Deng et al. investigates the progress in the application of aptamer-modified AuNPs in cancer marker detection and targeted therapy. Further, the authors also analyze the prospects and challenges associated with the advancement of AuNPs from the current basic research to clinical experiment.

Prostate-specific membrane antigen (PSMA) has been considered as a potential target for prostate cancer targeted diagnosis and therapy (Sun et al., 2021). Benefiting from the rapid development of nanobiotechnology, Xiao et al. constructs a tumor-targeted nanosystem with PA imaging and photothermal capabilities to co-deliver photosensitizer IR780 and paclitaxel for tumor therapy through surface modification, which uses perfluorocarbon as the core, polylactic acid-glycolic acid (PLGA) as the shell, PSMA as targeting molecules. Furthermore, under the guidance of ultrasound/PA imaging, the system can effectively combine photothermal and chemotherapy to accurately locate and kill prostate tumors.

The proliferation inhibition of a variety of cancer cells is mediated by hydroxyapatite nanoparticles (nHAp) and the demonstration of mechanism makes nHAp composites a hot Research Topic for prognostic materials for bone tumors (Shi et al., 2009; Zhang et al., 2019). Currently, many new functional nHAp composite materials have been shown to possess anti-tumor abilities and can effectively address postoperative bone defects, tumor recurrence, and metastasis. Herein, Zhang et al. presents a comprehensive overview of the application of nHAp in the treatment of bone tumors and explore the mechanisms responsible for nHAp’s ability to hinder tumor initiation and progression, and displayed future prospects for the development of nHAp materials for tumor therapy.

In summary, this series of related articles introduces the latest strategies and advances in functional biomaterial nanosystems in cancer treatment and tissue regeneration. *In vitro* and *in vitro* research and analysis of existing challenges will ultimately lead to a comprehensive understanding of translational and clinical applications of functional biomaterial nanosystems and point its future directions.
